# “I am very critical of my body, but I am not a worthless person”: A qualitative investigation of internalized weight stigma in Denmark

**DOI:** 10.3389/fpsyg.2022.1049568

**Published:** 2023-01-17

**Authors:** Emma Davidsen, Majken Lillholm Pico, Peter Sandøe, Thomas Bøker Lund

**Affiliations:** ^1^Health Promotion Research, Steno Diabetes Center Copenhagen, Herlev, Denmark; ^2^Department of Food and Resource Economics, University of Copenhagen, Copenhagen, Denmark; ^3^Department of Veterinary and Animal Sciences, University of Copenhagen, Copenhagen, Denmark

**Keywords:** internalized weight bias, internalized weight stigma, obesity, overweight, weight bias internalization scale, self-stigma, WBIS-2F

## Abstract

**Aim:**

The aim of this study was to explore how two of the main dimensions of internalized weight stigma (IWS), namely weight-related self-devaluation and distress, play out in the lives of people with excess weight (EW), and to study whether there are further dimensions of IWS.

**Method:**

Ten informants with EW were included in this study. The sample size was determined prior to data collection based on available resources at the time of data collection. All informants both participated in in-depth interviews and responded to the questionnaire WBIS-2F consisting of the two subscales: weight-related self-devaluation and distress. The interview accounts were thematically coded and compared with the informants’ scoring on WBIS-2F.

**Findings:**

Seven themes were identified from the in-depth interviews: (1) devaluation of competencies, (2) self-blame, (3) bodily devaluation, (4) ambivalence, (5) anticipated stigma, (6) coping strategies, and (7) mental well-being. Overall, the informants scored low on the WBIS-2F subscale weight-related self-devaluation and high on the subscale weight-related distress. The qualitative findings echo the informants’ scoring on WBIS-2F. However, novel aspects of IWS not covered by WBIS-2F were also identified. In particular, bodily devaluation presented itself as an integral part of IWS.

**Conclusion:**

The two current dimensions of WBIS-2F were retrieved, but important additional aspects of IWS were also identified. Future research is needed to evaluate and test both qualitatively and quantitatively whether the additional aspects of IWS identified in this exploratory examination are separate constructs of IWS.

## 1. Introduction

Weight-related stigma has been identified and indeed studied in countries across the world. Experienced weight discrimination has been found in almost all aspects of life: studies have found it in children, in educational institutions, in the labor market, in health care, in popular media, and in interpersonal relationships (Puhl and Heuer, 2009; Puhl et al., 2021). Interpersonal relationships, especially, have been identified as a primary source of experienced weight discrimination. Puhl et al. (2021) found that in an international study spanning across six countries, more than half of their respondents (55.6–61.3%) reported experiences of weight discrimination, with 87.6% confirming that the discriminators were family members. In addition, while stigma based on race, skin color, and sexuality seem to be decreasing, weight-related stigma appears to be on the increase (Charlesworth and Banaji, 2019). Experienced weight discrimination has been associated with both mental and physical adverse health outcomes, including depression, anxiety, high allostatic load, high levels of cortisol, and early death (Major et al., 2014; Puhl and Suh, 2015; Tomiyama et al., 2018). Studies have also linked weight discrimination with higher levels of internalized weight stigma (IWS) (Pearl et al., 2018; Puhl et al., 2018).

A common definition of IWS, also referred to as “weight bias internalization” and “self-directed weight stigma,” is yet to be agreed upon, and researchers within the field have recently called for a Delphi study to reach a consensus on the matter (Austen et al., 2021). In this study, however, IWS is defined as (1) awareness of negative stereotypes relating to one’s social identity as a person with EW; (2) agreement with these stereotypes; (3) the application of the stereotypes to oneself; and (4) self-devaluation because of one’s social identity (Durso and Latner, 2008; Pearl and Puhl, 2018).

Puhl et al. (2018) have indicated that 40% of Americans with EW report IWS. IWS, like enacted weight discrimination, seems to follow people with EW through childhood and into adulthood (Pearl and Puhl, 2018; Fields et al., 2021). Recently, Clark and colleagues also highlighted the potential negative impact social media – with more than four billion users worldwide – have on IWS by promoting certain ideal body types (Clark et al., 2021; Backlinko, 2022). Like experienced weight discrimination, IWS is associated with adverse health outcomes, both mental and physical (Pearl and Puhl, 2018). IWS appears to have been studied primarily in quantitative, cross-sectional studies. Durso and Latner’s (2008) Weight Bias Internalization Scale (WBIS-11) is used as the predominant measure here (Durso and Latner, 2008; Pearl and Puhl, 2018). Assuming that IWS not only includes an awareness of negative stereotypes to oneself but also a self-devaluation in response to one’s stigmatized identity, Meadows and Higgs argues that WBIS-11 does not adequately capture the multifaceted nature of IWS. In 2019, using the original pool of 19 items used to create and validate WBIS-11 in 2008, Meadows and Higgs proposed a revised version of WBIS-11 consisting of two factors: (1) weight-related distress and (2) weight-related self-devaluation (Meadows and Higgs, 2019). They constructed the revised version using exploratory and confirmatory factor analysis, and they labeled the novel scale WBIS-2F.

When constructing the two subscales, Meadows and Higgs found that two items measuring weight-related self-devaluation, namely “I hate myself for being overweight” and “My weight is a major way that I judge my value as a person,” loaded onto the weight-related distress subscale (Meadows and Higgs, 2019). They acknowledge this as a limitation of the WBIS-2F scale and question whether the original pool of 19 items incorporates all relevant aspects of IWS. Yet, with its two subscales, Meadows and Higgs argue that WBIS-2F scores reflect the different types of IWS more accurately and that WBIS-2F, therefore, offers more nuanced insights into the phenomenon of IWS (Meadows and Higgs, 2019).

The subscale of weight-related distress contains statements such as: “I feel anxious about being overweight because of what people might think of me,” “I wish I could drastically change my weight,” “Because I am overweight I do not feel like my true self” and “Because of my weight, I do not understand how anyone attractive would want to date me.” Hence this subscale refers not only to feelings of distress, annoyance, and frustration at one’s weight but also to the perceived lower worth or status of the distressed person’s body in society.

The subscale of weight-related self-devaluation contains statements such as: “I do not feel that I deserve to have a really fulfilling social life as long as I am overweight,” “I feel that being overweight does not interfere with my ability to be a good and decent person” (reverse coded), and “If other people do not treat me with respect, I should put up with it because of my weight.” This subscale addresses themes such as intelligence, being worthy of respect, and moral status.

Although WBIS-2F offers a more granular understanding of IWS, it is still not well understood how the two aspects, weight-related distress, and weight-related self-devaluation, play out in the everyday lives of people with EW. Nor do we know whether all important dimensions of IWS have now been recognized.

The qualitative study we report here was designed to contribute to filling these research gaps. Thus, its aim was to explore how weight-related distress and weight-related self-devaluation play out in the everyday lives of people with EW and to investigate whether IWS has potential additional dimensions on top of those recorded by WBIS-2F.

## 2. Materials and methods

### 2.1. Ethical considerations

Ethical approval for the study was obtained from The Research Ethics Committee for SCIENCE and HEALTH at Copenhagen University, Denmark (case no. 504-0269/21-5000). The aims and scope of the study were explained to the informants orally. All were told that the interviews would be recorded, and that excerpts from them would be included in the dissemination of results. Informed written consent was obtained prior to the data collection phase. Informants were informed that they could withdraw their consent at any time after data collection. It was emphasized that there were no right or wrong answers and that the purpose of the interviews was to understand their experiences and points of view. The interviewer adapted each interview to the terminology the informants used to describe their own bodies (e.g., fat, big-boned, overweight, obese) in an effort to ensure the interview would not cause offense. All transcripts were pseudonymized. As the topic of the interview was sensitive, no therapeutic questions, or questions that might challenge the informants’ self-perceptions, were included in the interview guide (Kvale and Brinkmann, 2009, p. 136).

### 2.2. Recruitment and inclusion of participants

We used a purposeful homogeneous sampling design (Robinson, 2014). The sample size was determined prior to data collection, as this study was originally a part of ED’s Master’s degree with pre-defined and limited time available. Further, since people with EW is a hard-to-reach population, we employed different recruitment routes. Informants were recruited from social media (*n* = 3), a closed network used by a fat activist interest group (“Fat Front”; *n* = 1), the Department of Bariatric Surgery at a Danish hospital in the Capital Region (*n* = 5), and snowballing (*n* = 1). Inclusion criteria for participation in the study were that the informants had a Body Mass Index (BMI kg/m^2^) >30, were aged 18 or above, and understood the text of a consent form written in Danish.

### 2.3. Participant composition

A total of 10 informants were included in the study. The final sample included eight women and two men. The informants ranged from age 31 to 57 with a mean age of 44 (SD 7.9). Four informants were in relationships. Five had completed medium-length tertiary education. All 10 informants had a BMI kg/m^2^ above 30, which is the standard threshold for obesity (Center for Disease Control, 2021). Half of the informants were on a waiting list for bariatric surgery (Table 1).

**Table 1:** Characteristics of informants.

Participant’s pseudonyms	Age group	Sex (Female/Male)	Marital status	Education	Employment status	BMI group	Waiting list for bariatric surgery	WBIS-2F	
								Self-devaluation (Range 1 to 7)	Weight distress (Range 1 to 7)
Mary	41–45	F	In a relationship	Long	Employed	46–50	No	1.0	3.0
Sara	46–50	F	Single	Middle	Employed	46–50	No	1.0	1.0
Jane	36–40	F	Single	Long	Employed	30–35	No	3.2	5.6
Cecilia	31–35	F	Single	Long	Self-Employed	36–40	No	1.1	5.0
Kirsten	41–45	F	Married/Cohabitant	Short	Employed	41–45	No	2.2	4.6
Christian	46–50	M	Single	Middle	Employed	46–50	Yes	2.2	3.7
Lea	56–60	F	Single	Short	Early retiree	36–40	Yes	2.8	6.4
Simon	31–35	M	Single	Short	Employed	51–55	Yes	3.0	6.0
Hellen	41–45	F	Married/Cohabitant	Middle	Employed	41–45	Yes	2.0	5.4
Hanna	51–55	F	Married/Cohabitant	Short	Early retiree	41–45	Yes	3.0	6.4

### 2.4. Procedure

The data for this study were collected using two measures: (1) non-standardized in-depth semi-structured interviews collecting qualitative data of the informants’ lived experiences, and (2) a standardized measure for IWS, namely the questionnaire WBIS-2F. The core of the study was conducted as part of ED’s Master’s degree in Public Health Science at the University of Copenhagen in 2019. The original focus of the thesis project was to investigate experienced weight discrimination as well as IWS. However, in this article, only data on the participants’ IWS will be presented.

### 2.5. In-depth interviews

Five of the interviews were conducted face-to-face in settings chosen by the informant (at the library, at two informants’ homes, and at an informant’s office). The remaining five interviews were conducted face-to-face at a Department of Bariatric Surgery, where the five informants involved were also recruited. No relationships were established with participants prior to the commencement of the study. The interviews were conducted in January and February 2019 and lasted 40–90 min each. All were conducted by the same interviewer (ED). Interviews were initiated with a short introduction, during which ED’s educational background was presented and the purpose of the interview was stated. Thereafter a semi-structured interview guide was used to conduct the interviews (see Supplementary material, Appendix A). All the questions in the interview guide, which was developed by ED, PS, and TBL, were formulated as open-ended questions. The semi-structured interview guide was designed to produce in-depth accounts of the informants’ experiences with both IWS and experienced weight discrimination. Thus, the questions focused first on general issues about the informants designed to get a sense of who they were as individuals and to establish a safe atmosphere in the room. Next, informants were asked about their general experience with weight, throughout childhood and as adults. Then, the questions focused on direct, indirect, and structural discrimination, and finally, questions were asked about the potential consequences of such discrimination. IWS was investigated throughout. However, specific questions about limiting oneself due to one’s weight were also asked in connection with the experienced consequences of experiencing stigma. In addition, the themes in the interview guide were guided by four testimonial statements on the Danish Fat Front’s website (Anon, 2019). The statements had originally been submitted by people with EW who had experienced weight discrimination or limitations because of their weight. In the interviews, the statements were read aloud, and the informants were then asked to give their initial reactions to them and comment on them. All interviews followed the same interview guide with follow-up questions and probing, where appropriate. The interview guide can be found in the Supplementary material, Appendix A.

### 2.6. Questionnaire data

Each informant completed the questionnaire WBIS-2F. WBIS-2F consists of two subscales, weight-related distress (7 items) and weight-related self-devaluation (6 items; Meadows and Higgs, 2019). The informants’ responses to WBIS-2F were scored in line with the scoring guides provided by Meadows and Higgs (2019). There is currently no defined cut-off value in WBIS-2F indicating whether a person with EW reports IWS. However, previous studies have categorized a score of 4.5 out of 7.0 on WBIS-11 as a high level of IWS, and this was therefore also applied in the present study (Puhl et al., 2018).

### 2.7. Data analysis

For the questionnaire data, the mean and standard deviation of the two subscales of WBIS-2F were calculated. All in-depth interviews were audio-recorded and subsequently transcribed verbatim by ED. An abductive thematic analysis was used to analyze the data (Braun and Clarke, 2006). During data collection, ED, PS, and TBL discussed the findings, focusing on their consistency with existing stigma theory, and on the identification of novel themes presenting complexities and nuances of IWS. When data collection was concluded and all the interviews had been transcribed, the transcriptions were imported and coded in QSR NVivo 12 (QSR International Pty Ltd., 2020). Data were coded abductively, identifying themes and subthemes that could add insights into the complexity of IWS. The abductive approach is useful in theory generation and refinement, as it can provide insight into novel themes in data, which in turn can provide insights and nuance to the existing theory (Thompson, 2022).

All transcripts were first individually coded by ED. After that, MP individually coded all the transcripts based on EDs suggested codes, while also looking for additional themes that were not covered by EDs initial coding. MP identified one additional theme, which all authors agreed was important to include. Based on the themes and subthemes identified by ED and MP a final codebook was developed, which all authors reviewed and agreed was appropriate. ED then revisited the transcripts with a view to systematically code for this added theme. After that, the authors assessed inter-rater reliability between ED’s and MP’s individual coding of the transcripts. The overall agreement was 93%, and Cohen’s Kappa was 0.85 (McHugh, 2012). In total, there were just 21 disagreements, and they were discussed and resolved jointly by ED and MP. Based on the themes and subthemes identified by ED and MP a codebook was developed. PS and TBL reviewed the codebook and following this, appropriate adjustments were made where relevant. In Table 2, we present all the themes and subthemes in the codebook, along with subtheme-specific Cohen’s kappa values and definitions (see Supplementary material, Appendix B for the full Table 2 with exemplifying quotes for each subtheme). The number of informants who reported on each theme is also shown in Table 2. As the study involved only 10 participants, it was not possible to generalize based on the informants’ WBIS-2F scores. Thus, the WBIS-2F scores were used descriptively to substantiate the qualitative data.

**Table 2:** Identified themes and subthemes presented with the Cohen’s kappa value indicating inter-rater reliability.

Theme	Subtheme (Kappa; −1 to +1)	Number of informants (1–10)	Definition
Devaluation of competencies	Competencies (0.375)	3	Devaluation of one’s own capabilities or competencies due to one’s weight
Self-blame	Self-blame (1.000)	6	The perception that the EW is ones’ own fault
Bodily devaluation	Beauty ideals (0.615)	9	Devaluation of ones’ body due to not meeting prevailing beauty ideals
	Romantic relationships (0.800)	4	Devaluation of ones’ body in relation to a romantic partner
Ambivalence	Ambivalence (0.583)	6	Reported discrepancies between the way the informants perceive themselves, and their bodies, as well as other people with EW
Anticipated discrimination	Assumptions of others’ thoughts (1.000)	8	Assumptions of what other people might think of them due to their weight
	Looks (0.412)	9	Reported anticipated discrimination based on certain looks given to the informants
Coping strategies	Reacting against stigma (0.200)	5	Reactions or active decisions to act against both internalized and experienced stigma
	Avoidance: Socially (0,800)	6	Avoiding or limiting social situations due to expected discrimination or internalized stigma
	Avoidance: Physical activity (1.000)	7	Avoiding or limiting being physically active due to expected discrimination or internalized stigma
	Avoidance: Wearing nice/revealing clothes (1.000)	5	Avoiding or limiting wearing revealing or nice clothes due to internalized beauty ideals
	Avoidance: Applying for job (1.000)	2	Not applying to jobs due to expected discrimination or lack of faith in own competences
	Avoidance: Travelling (1.000)	2	Avoiding or limiting travelling due to expected discrimination and/or internalized stigma
	Avoidance: Healthcare seeking (−0.154)	1	Not seeking healthcare due to expected discrimination
	Compensation: Work (0.800)	4	Feeling the need to compensate for EW and not confirming stereotypes by working harder
	Compensation: Social life (1.000)	2	Feeling the need to compensate for EW and not confirming stereotypes by being extra social and likable
	Compensation: Appearance (1.000)	1	Feeling the need to compensate for EW and not confirming stereotypes by paying extra attention to one’s appearance
Mental well-being	Anxiety (1.000)	2	Reported anxiety due to EW
	Social anxiousness (1.000)	7	Feeling anxious or uncomfortable about social situations due to weight-related distress
	Sadness (1.000)	2	Reported sadness due to EW
	Loneliness (1.000)	1	Reported loneliness due to EW
	Disordered eating (1.000)	4	Reported disordered eating due to EW distress
	Aggression (1.000)	1	Reported aggression due to EW distress
	Low self-esteem (1.000)	7	Reported low self-esteem due to EW

## 3. Results

### 3.1. Characteristics of the informants

The informants had a range of demographic backgrounds, which made it possible to produce broad insights into experienced IWS across differences in BMI, age, educational background, and marital status (Table 1).

The informants’ self-reported scores on WBIS-2F differed substantially both between the two subscales and between the informants (Table 1). No informant scored more than 4.5 on the weight-related self-devaluation subscale [with a mean score of 2.14 (SD 0.8) and a max score of 3.2 (scale range: 1.0–7.0)], suggesting that there was a relatively low level of weight-related self-devaluation among the informants. By contrast, however, seven informants reported a high degree of weight-related distress [with a mean score of 4.71 (SD 1.6)]. Scores on the weight-related distress subscale ranged from 1.0–6.4 (scale range 1.0–7.0).

### 3.2. Qualitative findings

Seven themes were identified in the qualitative data: (1) devaluation of competencies, (2) self-blame, (3) bodily devaluation, (4) ambivalence, (5) anticipated stigma, (6) coping strategies and, (7) mental well-being. These themes all relate to the informants’ experience with IWS. Weight stigma was omnipresent in the qualitative data and will not be elaborated on further in this study. It was the experience of all informants, however, that Danish society was still stigmatizing people with EW.

“[…] we [Danes] are very controlled and homogenous as a people, and we like to have control over everything, and a big body is an uncontrolled body, in a way… We want to have control over our lives and own property and so on… which is why I think it [a big body] is provocative [in the mind of most Danes].” – Cecilia

#### 3.2.1. Devaluation of competencies

Two of the 10 informants reported situations in which they had devaluated their competencies as a consequence of exposure to the stereotypes associated with EW. The informants reporting this did not believe that they were amoral or unintelligent because of their EW, although they sometimes doubted their own credibility. Jane, who worked in the field of physical activity, reported that she sometimes felt out of place, or incompetent, at her job because of her EW.

“It has definitely been my idea of not fitting in [at work] and my concerns about being skilled enough [at my job] that I could channel into some kind of insecurity about – well if I look like this, how can I stand here and present [my work]?” – Jane

Jane also had the highest score on the weight-related self-devaluation subscale (3.2 out of 7.0, Table 1). Most informants, however, mentioned that they knew that their EW did not reflect their competencies or their intelligence. When asked about this, Sara, with a weight-related self-devaluation score of 1.0, answered:

“I saw a TV show yesterday where there is a woman who says, ‘my work colleagues consider me stupid because I'm so big’ or ‘they tell me I am lazy because I could just pull myself together and lose weight’, and I just think, I would not even think those things about myself, because I know I am intellectually strong, so it is not because I am not smart.” – Sara

In short, some informants reported doubting their own competencies and credibility because of their EW, but most did not even entertain such thoughts.

#### 3.2.2. Self-blame

Six informants blamed themselves for their size, either indirectly or directly. Lea, a single mother of four, for instance, reported that she would try various diets and diet pills but was unable to stick with these diets. She said she perceived herself as weak-willed as she failed each time. Jane and Sara, who were both actively trying to lose weight, referred to the basic principle that what comes in must come out.

“First of all, it [weight gain] is due to the fact that you eat more [calories] than you burn, it is simple mathematics.” – Jane

When the role of genetics and the effect of society were brought up, they were referred to as a bad excuse. The informants argued that one had to take responsibility for one’s own weight. As Mary, a healthcare professional put it:

“I definitely feel that saying overweight is in your genes is a poor excuse. I mean, you don’t get fat by not eating enough, or eating sensibly. I don’t believe in that, and I honestly have the philosophy that people who are using that excuse should be sent to a deserted island and then they wouldn’t stay fat very long.” – Mary

Consequently, Mary, expressing her perception of people being personally responsible for their EW, pointed back to the fact that she blamed herself for her EW. The self-blame was also manifested in prejudice about other people with EW, where both Mary and Jane reported being provoked or annoyed by people with EW who ate ice cream or took public transportation, as opposed to eating an apple or riding a bicycle. Upon further consideration, these reactions were also reported by the informants when they reflected on how they perceived themselves and the inner dialogs they could have, blaming themselves for making poor choices where food and exercise were concerned.

#### 3.2.3. Bodily devaluation

Nine of the informants reported devaluation of their bodies connected with EW. Sara was one of the informants with the lowest reported weight-related distress and self-devaluation (Table 1). She did not, however, question the idea that EW was undesirable from the perspective of health and beauty. She disliked receiving complimentary comments from people when she had lost weight. However, she saw this as something she had to embrace because in her opinion being slim was more beautiful than having EW.

“I have to accept that people tell me it [weight loss] looks smashing. I even said it to my niece this weekend. She had lost 23 kg, and she looks damn good. It [the weight loss] has helped immensely [with her looks].” – Sara

Bodily devaluation was also identified in the ways some of the informants’ talked about their bodies in relation to romantic relationships. Jane, who was actively trying to lose weight, had no wish to seek romantic partners before losing weight, as she did not feel “at home” in her body yet. Lea felt unable to leave her orally abusive boyfriend because she did not believe that, with her weight, anyone else would be interested in her sexually. Another informant, Hellen, did not feel sexually attractive to her husband.

It was Hanna who reported devaluating her own body as an adult in the most direct terms. Like many of the informants, Hanna had experienced dramatic fluctuations in body weight, and her experience after one episode of weight loss underlined how degradingly she had thought of her body during the periods in which she had EW.

“When I only weighed 70 kg, it was really nice to go out and buy clothes and be with people, because at that time I was not afraid to be with others, because I was pretty, right? And I had to get used to my body, that it had become smaller, and I would still sometimes think ‘Oh no, I am fat. I can’t do that’. So, it took some time before I got used to it.” – Hanna

#### 3.2.4. Ambivalence

A theme identified in six interviews was ambivalence. The ambivalence the informants reported concerned the way they perceived themselves, and their bodies, as well as other people with EW. It created frustration at times, and in some cases anger, alongside a need, or at least a desire, to fit into society.

“I'm like ‘Fuck you! Fuck your society!’, but I also feel like ‘Oh I am so wrong and believe everything I am told’. And I also have this rage in me which has also made me do exactly what I want.” – Cecilia

While Cecilia was very outspoken and reflective about her ambivalence, the mixed feelings of other informants were revealed less directly during the interviews.

Ambivalence can, for instance, be detected in Hellen’s outlook. Hellen was on the waiting list for bariatric surgery. She had a blog promoting health at every size and worked in the healthcare sector. She wanted to embrace all bodies as good bodies and believed that the stigma of EW was unjust. Meanwhile, she also felt that she was not sexually attractive and she did not want to exercise in her neighborhood or appear sweaty or exhausted at work, as she was embarrassed by her EW. The decision she had made to have bariatric surgery was made primarily on health grounds.

Another informant who was ambivalent about her EW was Mary. Mary also worked in the healthcare sector. She reported having high self-worth and overall satisfaction with her life. Despite this, she had a hard time identifying with her body.

“I also have some prejudices about being fat myself – that is, the fact that it is rarely upper-class people that are fat. After all, it is often social class 5 where they are. So sometimes I think about it myself. I'm a well-educated woman, and I have some healthy values and stuff like that, and I know what it takes to lose weight, but I still fall into that category, with the fat ones. And I can’t help but question myself. If I can’t even control my own weight, then what kind of human am I?” – Mary

The ambivalence disclosed in the informants’ narratives about living with EW highlighted the multifaceted nature of IWS in the informants.

#### 3.2.5. Anticipated discrimination

All the informants mentioned anticipated discrimination when they responded to questions about experiencing discrimination. Anticipated discrimination involves assumptions about what someone expects other people to think of them, or how others will react to them, given someone’s weight. Anticipated discrimination was described as an abiding concern of the informants in their everyday lives. Simon compared the anticipated discrimination he experienced in his adult life with the bullying he had endured during his school years.

“If something goes wrong when I walk by, or something happens, then I can see them standing and pointing and telling each other about him over there, it's him who takes up two seats in the bus, or it's him who eats more, and it’s him that destroys the world. I can see that, and then I stuff music in my ears, so I do not have to listen to them.” – Simon

Mary also described how she anticipated that her boyfriend would have issues with her weight because he had never dated anyone her size before. She often expected that any issues that might arise in their relationship would be about her weight, although in fact, this had never been the case. She feared meeting and socializing with her boyfriend’s friends, as she anticipated that they would think negatively about her because of her weight. The anticipated stigma here was not reported as something associated with experiences of actual discrimination. Rather it was regarded as the result of the informants’ awareness of the stigma attached to having EW and, in some cases, their own prejudice toward people with EW. The constant anticipation of being discriminated against created a need to compensate, and to have strategies in place allowing the informant to avoid situations where discrimination might occur.

#### 3.2.6. Coping strategies

The informants all reported having coping strategies to avoid potentially stigmatizing situations. Even the informants who had never experienced that their expectations of being discriminated against were confirmed continued to carry the anticipation of discrimination around as a constant fear. This also made them feel the need to compensate for their EW. Five informants said that they felt they had to compensate for their EW by overachieving in social or professional situations.

“I graduated 20 years ago, and I actually think I have felt the need to do extra work all of my professional life because I’ve anticipated that people think I am lazy due to their prejudice [toward people with EW]. I simply cannot live with that prejudice, because I really feel like I have done a lot of extra work, compared to some of my colleagues. Maybe I would be lazier if I was normal weight (laughs).” – Kirsten

All the informants said they used avoidance strategies to limit feelings of humiliation or degradation resulting from others’ reactions to their EW. Kirsten, for instance, reported not wanting to wear short or revealing clothes during the summer.

“I don’t find it super fun to change with other people by the public pools, or wear bathing suits, so in that way, I feel limited. […] I don’t want to show my body, so it has been an extremely long and hot summer when one prefers wearing a lot of layers” – Kirsten

In her attempt to try to avoid being confronted with the stigma associated with EW, Kirsten thus spent an entire summer feeling very hot and uncomfortable.

Other strategies reported by the informants included avoiding social situations, physical activities, and certain clothes, and applying for jobs, travelling and seeking medical help – all to evade potentially stigmatizing situations. Below, Hanna clarifies that she skips company parties as an avoidance strategy.

“I have tried to be at company parties, but I do not bother anymore. It is also because of the weight, that I think, ‘No, I do not know what I should wear’. And my colleagues, I mean of course they see me every day, but it's in my work clothes, and I just feel more exposed when I put on my own clothes.” – Hanna

Below, Helle describes that she experiences a public and neighborhood gaze at her body when exercising and that she avoids taking the stairs at work to avoid disclosing to her colleagues what she experiences as her failed body.

“I can actually feel a little inhibited when it comes to exercising outside. If I exercise outside, people in the area know me, and I do not want them to see me out of breath, so when I go [exercising] I try to go where there aren’t many other people. So, I feel a little inhibited by that. I also do not take the stairs at work, because I would not want to meet my colleagues [while walking up the stairs], because then they would see me about to cough my lungs out because I am overweight and therefore cannot take the stairs. It makes me feel exposed.”- Hellen

Another way the informants coped with the stereotype was by reflecting on it and reacting to it. In this respect, five of the informants reported being very aware of the prejudice associated with EW, and some were aware of their own IWS. They indicated that they felt the stereotypes were unjust. One informant, Cecilia, was actively trying to reclaim the word “fat.” As Cecilia put it, “I have chosen to come out as a fat person” – by which she meant that she was no longer ashamed to refer to herself as fat, as this was now, to her, a mere adjective without negative connotations. Cecilia was active in a pro-fat activist network. Three of the five informants were on the waiting list for bariatric surgery, and one was actively trying to lose weight. According to the informants wanting to lose weight, this desire was rooted mainly in a wish to be healthier, not in a sense that their EW was something to be ashamed of or a signal of personal limitations. Most of the informants who reported reflecting on, and reacting to, anticipated or actual stigma did not limit their lives and behavior to the same extent as the others.

“I think I used to be very withdrawn socially due to my weight, […] so I went into therapy, […] and since then I have actually chosen that I won’t be limited by anything.” – Christian

The remaining five informants, however, had not questioned or reacted to the stigma or their coping strategies. They devalued their bodies to the extent that they accepted that the society and culture they were part of were not made for people with EW, and they felt a need to avoid being seen or noticed.

#### 3.2.7. Mental well-being

Finally, nine informants reported that living with a stigmatized body had a negative effect on their mental well-being. The negative effects reported were anxiety, feeling uneasy or anxious in social situations, loneliness, disordered eating, violence, and low self-esteem.

Two informants reported having been diagnosed with anxiety. Cecilia, for example, had thanatophobia – an intense fear of dying – as she was afraid her death would cause a scene and draw attention to her EW.

“I have had quite a lot of anxiety about dying. For example, if I am in the cinema, I can get scared that I am dying […] but I am not actually afraid of death, I am afraid of causing a scene. Then I think to myself – okay, if I am dying, I just have to die quietly, so no one will notice. Then they can carry me out after the movie.” – Cecilia

Another reported form of anxiousness mostly related to feeling uneasy or uncomfortable in social settings due to EW. An example of this was Hanna, who refrained from going to large social gatherings, as she was scared what other people might think of her EW.

“If I am invited to a big party where I don’t really know people then I think ‘Oh no, I don’t want to go’ because I feel like everyone is going to think ‘Oh my!’ [when they see me]. So, no, no, I don’t do that either.” – Hanna

In other cases, informants reacted aggressively toward those who had harassed them.

“I was arrested once because I resorted to violence [because of being verbally harassed about body weight]. I talked to the police, and after hearing both sides of the story they [the police] dropped the charges. I can understand why he reported me to the police because I did resort to violence against him, but then again, he started it. So yes, it has been tough to have to look the other way, no matter what. It is easier to resort to violence and knock him out.” – Simon

## 4. Discussion

The informants expressed devaluing their bodies and blaming themselves for their EW. They were ambivalent about their bodies, their self-worth, and their identity. The ambivalence identified in the study indicates that the informants, from a detached point of view, believed that the prejudice and discrimination they had experienced because of their EW was morally wrong. However, their own IWS made it difficult for them to fully free themselves from the stigma associated with EW, even though the stigma was often only anticipated.

Coping strategies - such as avoidance behavior causing the informants to self-isolate, avoiding physical activity, and the avoidance of engagement with health care - and negative effects on mental well-being - such as anxiety, disordered eating, and aggression - were identified. These findings are in line with associations, already identified, between IWS and adverse health outcomes (Pearl and Puhl, 2018). The coping strategies identified also shed light on the positive effects of coming to terms with, and accepting, one’s own EW. The informants who reported that they accepted their bodies, or were actively trying to, adopted fewer avoidance strategies in comparison with those who did not accept their bodies. On the other hand, informants who were coming to terms with their bodies did not differ from the other informants in terms of mental well-being. This could be because their mental health issues were rooted in experiences in childhood, or at any rate from some period before they actively decided to accept their bodies. In this study, it was also discovered that accepting and embracing one’s EW does not exclude seeking bariatric surgery or attempting weight loss, as the informants who accepted or embraced their EW were still concerned about their long-term health.

Importantly, the negative effects of IWS on the informants’ mental well-being, including low self-esteem and disordered eating, were not reflected in the informants’ scores on the subtheme of weight-related self-devaluation, as no informant reported higher scores than 3.2 out of 7.0. This could indicate that the adverse mental health outcomes observed in the qualitative findings of this study do not stem from self-devaluation but rather are a consequence of weight-related distress and possibly other dimensions of IWS not yet fully captured in the quantitative measures of IWS.

The themes and subthemes identified in this qualitative study highlight the complexities of IWS, and the way in which IWS affects people with EW in their everyday lives. The study’s findings serve to support the assumption, made by Meadows and Higgs, that although WBIS-2F measures the complexities of IWS better than the original scale WBIS-11, it might not capture all the important dimensions of IWS (Meadows and Higgs, 2019).

During our nuanced investigation of IWS, it became clear that the informants expressed the same duality as that represented in WBIS-2F, since they scored very differently on the two subscales of weight-related self-devaluation and weight-related distress. According to its definition, IWS involves awareness, agreement, and the application of stereotypes leading to self-devaluation. Thus, according to the definition, self-devaluation should be present in cases of IWS. However, the informants in this investigation reported having IWS in the qualitative data even though they did not, for the most part, report self-devaluation on the WBIS-2F scale. Reported sadness, social anxiousness, assumptions about the thoughts of others, and withholding in romantic relationships were all identified themes and subthemes in the qualitative data, overlapping with items in the WBIS-2F subscale weight-related distress. Meanwhile, the WBIS-2F subscale weight-related self-devaluation was less represented in the qualitative data and only the identified theme of questioning one’s competencies seemed to be represented. Important aspects of IWS that we identified in the in-depth interviews, and that are not covered in WBIS-2F, were themes such as bodily devaluation in terms of beauty ideals, self-blame, ambivalence, anticipated discrimination, coping strategies, and mental well-being.

In short, then, our study findings suggest that the two sub-dimensions of WBIS-2F do not fully capture the complexity of IWS. Importantly, the study also suggests that IWS had an impact on the informant’s mental well-being, and that the ensuing coping strategies were the most burdensome aspect of EW. These employed coping strategies are not recorded by WBIS-2F, but they are important aspects of IWS.

Since we conducted the analysis for this study, Meadows and Higgs (2020) carried out an international cross-sectional survey where they examined and discussed the dimensional coverage of the original WBIS-11 further as developed and validated by Durso and Latner (Durso and Latner, 2008). Meadow and Higgs’ find that the respondents reported a general body-related self-judgment, rather than a generic self-devaluation (Meadows and Higgs, 2020). The body-related self-judgment was also found to mediate the relationship between weight-related distress and disordered eating to a higher degree than self-devaluation *per se* (*ibid*). We believe that Meadow and Higgs’ suggestion of body-related self-judgment converges with the findings about bodily devaluation in the present study. To the extent that they are similar (or at least, partially similar), our findings can be seen as providing an in-depth account of how body-related self-judgment plays out in the real life of people with excess weight.

### 4.1. Strengths and limitations

A clear strength to this study is that it is the first investigation to cast light on possible extra dimensions of IWS that do not seem to be captured by the new scale, WBIS-2F, by means of in depth-interviews.

The limits to this study include the small sample size. This means that the experiences reported in the interviews, and indeed the WBIS-2F scores, cannot be generalized, or extrapolated, to represent the lived experiences of all people with EW. This is a widely recognized limitation of qualitative research. The informants in this study, however, represented both different socioeconomic backgrounds and different age and BMI groups. Even though the study included a wide range of demographic types, it is of course possible that the people volunteering for the study differ from those who did not volunteer, introducing the possibility of selection bias.

Another limitation of this study is that the sample size was determined based on the limited resources available at the time of data collection, and not on data saturation. The nature of this study is thus exploratory and more research within this field, both qualitatively and quantitatively, is strongly encouraged.

We are aware that IWS is an abstract and complex phenomenon to investigate. This study thus serves as a contribution in that it sheds light on the lived experiences of people living with EW. We also acknowledge that the findings in this investigation are contextual and were shaped and produced in a collaboration between the interviewer and the informant.

The informants also exhibited a range of political opinions, with one, for example, belonging to a pro-fat activist group. The inclusion of this informant added experiences and opinions that are characteristic of activism, revealing phrases such as “coming out as fat.” It was considered important to include an informant with an activist agenda, as this represents a group of people with EW in Denmark. The study was also conducted in the capital region of Denmark. This means its findings do not necessarily reflect lived experiences and actions of people with EW in other parts of the country, or beyond Danish shores. Indeed, Denmark has a relatively low prevalence of people living with EW (18.5%), compared to countries such as Germany (26%), the United Kingdom (30%), and The United States (37%; Den Nationale Sundhedsprofil, 2021; Puhl et al., 2021). Puhl et al. (2021) did not, however, identify significant differences in experienced weight stigma in their international investigation into experienced weight stigma in countries with a varying prevalence of people living with EW. In addition, we believe it to be a strength of our study that our findings regarding the importance of bodily-devaluation echo Meadow and Higgs’ international investigation with respondents from 16 different countries (Meadows and Higgs, 2020). Lastly, it must be acknowledged that all the informants were Caucasian and that all were born and raised in Denmark. That made it impossible to pick up on cultural differences.

### 4.2. Future research

Although Meadows and Higgs managed to devise a more nuanced measure of IWS, their suggested scale, WBIS-2F leaves various questions about the multifaceted nature of IWS and how this is perceived by people with EW unanswered. The seven themes identified in this study suggest that WBIS-2F might be elaborated further as a scale for measuring IWS. Bodily devaluation was found to be a significant theme in the interviews. It is separate from weight-related self-devaluation, and we argue that it should be investigated further whether bodily devaluation can be considered as an independent third dimension of IWS. We therefore speculate but cannot examine this further based on the current data, that bodily devaluation may be highly involved in the mental processes that prompt people to develop coping strategies, and experience mental and social well-being challenges. Further, we find it likely that bodily devaluation may play an indirect role in the abovementioned outcomes through the anticipated stigma and experienced ambivalence instigated by this devaluation. We propose investigating this quantitatively in future research.

The ambivalence among the informants involved in our study – who felt that prejudice and discrimination toward people with EW were unjust, but still wanted to fit into society and thus abide by existing beauty and body ideals – calls for further investigation. As Meadows and Higgs point out, at least two of the seven items on the weight-related distress subscale refer to self-devaluation: “I hate myself for being overweight” and “My weight is a major way that I judge my value as a person.” This overlap of items between the two subscales might indicate that the people affected by IWS feel ambivalent about their bodies and self-worth because of their weight. In the interviews, ambivalence also seemed to be an indicator of IWS, which suggests it ought to be investigated further and could possibly be included in a reassessed version of WBIS-2F. Further, the overlap of items between the two sub-scales, coupled with the additional themes identified in this study, indicate that future research ought to revisit the conceptualization of IWS. We have argued that a more granular identification of latent dimensions – specifically, bodily devaluation and, perhaps, ambivalence – is relevant to the understanding of IWS in everyday life and its possible consequences in terms of coping and mental well-being.

Future research should also consider how people with EW experience and are affected by IWS in different cultural settings. Although Brewis and Wutich found weight stigma to be a global issue (Brewis and Wutich, 2015) and international studies have identified very few differences in experienced weight discrimination between countries (Puhl et al., 2021) there are still important cultural aspects relevant to IWS that ought to be investigated further. In an investigation by Agyapong et al. (2020), they found that Ghanaians associated EW with wealth and fertility, and Draper and colleagues found that some South African women consider EW to be more attractive and believe that being slender is associated with illnesses such as tuberculosis or HIV/AIDS (Draper et al., 2015). These findings indicate that IWS might be experienced and reported differently in countries with positive connotations about EW than in countries where EW primarily is perceived negatively.

The findings in this study also highlight some of the potential conceptual issues with the definition of IWS. As the definition of IWS also encompasses self-devaluation, something that can be viewed as a consequences of IWS, i.e. having low self-esteem, can also be viewed as a component of IWS. We, therefore, concur with Austen and colleagues that a consensus on how to define IWS is needed to properly investigate IWS and the impact it has on people’s lives (Austen et al., 2021). To shed light on the conceptualization of IWS and potential implications hereof, we propose conducting a similar study to ours, but with a significantly larger sample size, ensuing a larger variety in demographic characteristics and cultural representation, employing mixed methods to validate the quantitative findings with qualitative insights.

Finally, the informants in this study reported that IWS had had a huge impact on their day-to-day lives, especially in terms of their development of coping strategies and their experience of adverse impacts on their mental well-being. This highlights the importance of investigating not just the degree to which people with EW report IWS but also what the implications of IWS are in the lives and the well-being of those displaying it.

## 5. Conclusion

The present exploration of the concept and measurement of IWS using qualitative interview data underlined the complexity of IWS. The participants in the study, all with EW, reported varying degrees of IWS and had an overall tendency to report a high degree of weight-related distress and a relatively low degree of self-devaluation due to their weight. This investigation also emphasizes aspects of IWS not captured by WBIS-2F. Specifically, it adds to the concept of IWS in two novel ways. First, issues such as ambivalence and coping strategies, that are not embedded in the measurement of IWS, are still important aspects of it. Second, the results emphasize that people with EW may devalue their bodies despite not reporting self-devaluation in terms of having poor morals or low intelligence. In addition, this study adds to the conceptualization of IWS, as it was found that the participants in the study did have IWS which impacted their everyday lives even though they did not suffer from general lack of self-evaluation.

These findings support, on the one hand, the existence of two dimensions of IWS, as suggested by Meadows and Higgs (2019): weight-related self-devaluation and weight-related distress. However, the identified nuances may provide important insights of value for future scale development or refinement. It is important to note that this study is exploratory, which is why we propose that future research should consider exploring these additional dimension of IWS both qualitatively and quantitatively, since IWS and all its nuances and implications still is far from fully understood.

## Data availability statement

The data presented in this article cannot be shared as the participants did not give their informed consent to share their responses. Requests regarding the datasets should be directed to the first author.

## Ethics statement

The studies involving human participants were reviewed and approved by The Research Ethics Committee for SCIENCE and HEALTH at Copenhagen University, Denmark (case no. 504–0269/21-5000). The patients/participants provided their written informed consent to participate in this study. Written informed consent was obtained from the individual(s) for the publication of any potentially identifiable images or data included in this article.

## Author contributions

ED, PS, and TL designed the study. ED collected data and wrote the first manuscript draft. ED and MP analyzed data. PS and TL provided critical feedback on the study and qualitative analysis. All authors participated in editing and reviewing the manuscript and all authors approved the final draft.

## Conflict of interest

ED and MP are currently employed at Steno Diabetes Center Copenhagen, a public hospital and research institution in the Capital Region of Denmark which is funded partly by a grant from the Novo Nordisk Foundation. PS regularly collaborates with employees of Novo Nordisk on subjects other than those covered in this paper. PS and TL have previously received a grant from Novo Nordisk to study the attitudes of GPs toward people with overweight.

## Publisher’s note

All claims expressed in this article are solely those of the authors and do not necessarily represent those of their affiliated organizations, or those of the publisher, the editors and the reviewers. Any product that may be evaluated in this article, or claim that may be made by its manufacturer, is not guaranteed or endorsed by the publisher.
