# Supplementary material for: “I am very critical of my body, but I am not a worthless person”: A qualitative investigation of internalized weight stigma in Denmark
Source: Front Psychol. 2023 Jan 17;13:1049568. doi: 10.3389/fpsyg.2022.1049568 (PMC9886864; doi:10.3389/fpsyg.2022.1049568)
Supplement: Supplementary file 1 [file Data_Sheet_1.docx]

**Supplementary material**

**Appendix A: Interview guide**

| **Focus area** | **Questions and prompts** |
| --- | --- |
| General introduction | What is your name, age, educational background, job title, origin?  What do you like to do in your spare time?  How was your upbringing and your family life? |
| Weight and body in general | How do you perceive your body in general?   - *[Prompt] Are you happy with it? Do you feel like it is limiting you?*   Have you considered why you have the weight you do?   - *[Prompt] Do you know how it happened? Did you have high weight as a child? Does it run in the family?*   Is your weight, in general, a problem for you?   - *[Prompt] Does it affect you in your everyday life? If so, how?*   Have you ever been bullied or teased due to your weight?   - *[Prompt] If yes, is it something that has affected your social life as an adult?*   Do you wish to lose weight?   - *[Prompt] If yes, how? Is it something you have tried?* - *[Prompt] if no, have you experienced that someone else wanted you to lose weight?* |
| Direct discrimination | Quote*: “My finger had been hurting for a long time and eventually, I could not bend it at all, so I went to the doctor. The first thing she asked me to do was step on the scales. I protested and told her that this was only about my finger and that I had an eating disorder and had not weighed myself for six years and it would trigger an episode. But she insisted and then spent 15 min. telling me about various diets I could go on. I just sat there, I was in shock and could not say anything.”*  What are your thoughts about this quote?   - *[Prompt] Is it something you recognize? If yes, how?*   Have you ever experienced that someone addressed your weight in a setting or situation where you found it inappropriate?   - *[Prompt] If yes, what happened? How did you react? Was anyone else there at the time and how did they react? Is it something you have discussed with someone after?*   Have someone in your social circles ever addressed your weight?   - *[Prompt] If yes, what did they say? How did they say it? How did it make you feel?* |
| Indirect discrimination | Quote: *“I usually have music in my ears when I go outside, because then at least I only have to be confronted with peoples’ looks.”*  What are your thoughts about this quote?   - *[Prompt] Is it something you recognize? If yes, how?*   Have you ever experienced situations where you felt you were not heard or seen, due to your weight?   - *[Prompt] If yes, can you describe the situation? How did you react? Has it affected how you present yourself or act in similar situations?*   [If they experience discrimination in general]  How do you usually react if you are upset with something someone had said or done to you? |
| Structural discrimination | Quote: *“I never go to the cinema or anything like that and never travel. The seats are simply not big enough and the seat belt in the plane is too small”*  What are your thoughts about this quote?   - *[Prompt] Is it something you recognize? If yes, how?*   Have you ever experienced that you did not feel comfortable somewhere due to physical limitations?   - *[Prompt] If yes, can you describe the situation? How did it make you feel?*   Have you ever experienced that a clothing store did not carry your size?   - *[Prompt] If yes, can you describe the situation? How does it make you feel?* |
| Consequences & internalised weight stigma | Quote: “*I have a little trouble exercising because I feel stared at every time I go to the gym or try to run outside.”*  What are your thoughts about this quote?   - *[Prompt] Is it something you recognize? If yes, how?*   Is there something you wish to do, but you feel that your weight is preventing you from?   - *[Prompt] If yes, in what way?*   Are there things you do not do, or have stopped doing, due to your weight?   - *[Prompt] What are the barriers you experience in regard to doing it? How come? Does it stem from previous experiences? What do you expect will happen if you did it?*   Do you feel that your weight is a hindrance to your social life?   - *[Prompt] When do you get that feeling?*   Is there something you imagine would change in your life if you weighed less?   - *[Prompt] If yes, in what way?* |

**Appendix B: Table 2 - Full overview of themes, subthemes, inter-rater reliability and quotes**

| **Theme** | **Subtheme**  **(Kappa; -1.00-1.00)** | **Nr. of informants (1-10)** | **Definition** | **Example** |
| --- | --- | --- | --- | --- |
| Devaluation of competencies | Competences  (0.375) | 3 | Devaluation of ones’ own capabilities or competences due to ones’ weight | “It has definitely been my idea of not fitting in [at work] and my concerns about being skilled enough [at my job] that I could channel into some kind of insecurity about – well, if I look like this, how can I stand here and present [my work]?” – Jane  “I don’t feel that I am good enough at my job. I really want to please everyone, both my management and my employees, I want everyone to like me and that of course also has to do with my overweight.” - Hellen |
| Self-blame | Self-blame  (1.000) | 6 | The perception that the EW is ones’ own fault | “First of all, it [weight gain] is due to the fact that you eat more [calories] than you burn, it is simple mathematics.” – Jane  “I get why people blame everything else than themselves [for their EW], because it is really easy […] We have given people every possibility to make up excuses not to do the right thing, and then we, as people, quickly blame everything but ourselves. I am a fan of taking responsibility for your own actions.” – Jane  “I definitely feel that saying overweight is in your genes is a poor excuse. I mean, you don’t get fat by not eating enough, or eating sensibly. I don’t believe in that and I honestly have the philosophy that people who are using that excuse should be sent to a deserted island and then they wouldn’t stay fat very long.” - Mary |
| Bodily devaluation | Beauty ideals  (0.615) | 9 | Devaluation of ones’ body due to not meeting prevailing beauty ideals | “I have to accept that people tell me it [weight loss] looks smashing. I even said it to my niece this weekend. She had lost 23 kg, and she looks damn good. It [the weight loss] has helped immensely [with her looks].” – Sara  “When I only weighed 70 kg, it was really nice to go out and buy clothes and be with people, because at that time I was not afraid to be with others, because I was pretty, right? And I had to get used to my body, that it had become smaller, and I would still sometimes think ‘Oh no, I am fat. I can’t do that’. So it took some time before I got used to it.” – Hanna |
|  | Romantic relationships  (0.800) | 4 | Devaluation of ones’ body in relation to a romantic partner | “I can get really upset. For instance, my boyfriend, who I met four years ago, he has only had really slim girlfriends before me, and when we met each other, I wasn’t as big as I am now. Since we met each other I have gained, well, around 40 kg. and that is tough on me” - Mary  “My sex life isn’t the same as I imagine it would be if I had a normal body and that is because the norms set by society define what is considered sexy and attractive and that is something I still really struggle with.” - Hellen |
| Ambivalence | Ambivalence  (0.583) | 6 | Reported discrepancies between the way the informants perceive themselves, and their bodies, as well as other people with EW | “I'm like ‘Fuck you! Fuck your society!’, but I also feel like ‘Oh I am so wrong and believe everything I am told’. And I also have this rage in me which has also made me do exactly what I want.” – Cecilia  “To this day I have to admit I can get a little angry if I see overweight people take the bus […] I mean I have almost always been riding my bicycle everywhere, but I feel like you have a choice […] If you rode your bicycle the 100 or 1000 meters you had to go [with bus] that would be way healthier for you. I mean, just do it. [*Interviewer*: Do you think this has something to do with how you feel about yourself?] Yes, definitely […] I have been telling myself that I bike everywhere, I walk and exercise regularly […] so sometimes the body I saw in the mirror didn’t really fit with how I perceived myself, and I could be really cruel to myself or feel disconnected from myself.” – Jane  “I also have some prejudices about being fat myself – that is, the fact that it is rarely upper-class people that are fat. After all, it is often social class 5 where they are. That is, whether it is ignorance or what it is that makes one fat. So sometimes I think about it myself. I'm a well-educated woman, and I have some healthy values ​​and stuff like that, and I know what it takes to lose weight, but I still fall into that category, with the fat ones. And I can’t help but question myself. If I can’t even control my own weight, then what kind of human am I?” - Mary |
| Anticipated discrimination | Assumptions of others’ thoughts  (1.000) | 8 | Assumptions of what other people might think of them due to their weight | “If something goes wrong when I walk by, or something happens, well then I can see them standing and pointing and telling each other about him over there, it's him who takes up two seats in the bus, or it's him who eats more, and it’s him that destroys the world. I can see that, and then I stuff music in my ears, so I do not have to listen to them.” – Simon  “[On the bus] They probably think ‘Come on, why does her fat body have to be in my way?’“ – Lea  “I don’t want to be seen in such a place [fast food place]! I will not be exposed to ridicule, I mean, you feel very exposed as a fat person […] You will never see me in a McDonalds or something like that. Over my dead body. Then I will be seen as the fat girl eating burgers.” - Cecilia |
|  | Looks  (0.412) | 9 | Reported anticipated discrimination based on certain looks given to the informants | “I often listen to music because I feel like I can be by myself, so to say, and then I can ignore that someone is making weird faces at me, and I can just get on with my day and think ‘fuck him or her’ and move on.” – Simon  “Random people’s looks don’t really affect me but looks from people who I have wanted to have some sort of relationship with matter. Or I mean, they might just see me for who I am, but I imagine how they must see me and that has been awful.” - Jane |
| Coping | Reacting against stigma  (0.200) | 5 | Reactions or active decisions to act against both internalised and experienced stigma | “I am… I guess you can say, coming out as fat, because I have been living my whole life pretending not to be fat. It sounds weird because of course I have been fat all of my life, but I have done so many things to make sure people didn’t notice my size because I was scared to lose the privileges I felt I had despite my weight. So, these last couple of years I have been really engaged with this issue.” – Cecilia  “I think I used to be very withdrawn socially due to my weight, […] so I went into therapy, [...] and since then I have actually chosen that I won’t be limited by anything.” – Christian |
|  | Avoidance:  Socially  (0,800) | 6 | Avoiding or limiting social situations due to expected discrimination or internalised stigma | “[*Interviewer*: Do you feel like it [your body] limits you socially?] Yes, because I don’t want to go out, I don’t want to do anything. I only really want to go out with my children and then I have one friend who I also go out with sometimes.” – Lea  “I have thought a lot about my weight in relation to meeting my boyfriend’s friends, the ones who don’t already know me, because maybe they saw me when we started dating, and now that I weigh much more I don’t really want to see them, or at least I contemplate it a lot.” – Mary  “I have tried to be at company parties, but I do not bother anymore. It is also because of the weight, where I think, ‘No, I do not know what I should wear’. And my colleagues, I mean of course they see me every day, but it's in my work clothes, and I just feel more exposed when I put on my own clothes.” – Hanna |
|  | Avoidance: Physical activity  (1.000) | 7 | Avoiding or limiting being physically active due to expected discrimination or internalised stigma | “It has become more difficult as I have become older to find a type of exercise that I enjoy. I used to swim a lot and I really enjoyed it […] but now I don’t swim anymore because I don’t want to go to the public pools.” – Cecilia  “I can actually feel a little inhibited when it comes to exercising outside. If I exercise outside, people in the area know me, and I do not want them to see me out of breath, so when I go [exercising] I try to go where there aren’t many other people. So, I feel a little inhibited by that. I also do not take the stairs at work, because I would not want to meet my colleagues [while walking up the stairs] because then they would see me about to cough my lungs out because I am overweight and therefore cannot take the stairs. It makes me feel exposed.”- Hellen |
|  | Avoidance:  Wearing nice/revealing clothes  (1.000) | 5 | Avoiding or limiting wearing revealing or nice clothes due to internalised beauty ideals | “I don’t find it super fun to change with other people by the public pools, or wear bathing suits, so in that way I feel limited. […] I don’t want to show my body, so it has been an extremely long and hot summer, when one prefers wearing a lot of layers.” – Kirsten |
|  | Avoidance:  Applying for job  (1.000) | 2 | Not applying to jobs due to expected discrimination or lack of faith in own competences  ‘ | “I have some career dreams, some things I am contemplating about my future that I want to do, and as I look right now I don’t see myself fitting in [to that work environment] physically.” – Jane  “I wouldn’t say that I limit myself [due to my weight], but it’s not like I don’t think about it either, for example in relation to applying for another job.  [*Interviewer*: Can you elaborate on this?]  “I think people hold a lot of prejudice against [people with EW]. I mean, people think big people are really lazy […] and even though I had good grades [in school] I was one of the last people to be employed.” – Kirsten |
|  | Avoidance: Travelling  (1.000) | 2 | Avoiding or limiting travelling due to expected discrimination and/or internalised stigma | “I mean, I sometimes travel, but when I am sitting in the seat [on the plane] my butt pokes out the sides and then if someone has to sit next to me who is also a little heavy, we can definitely feel each other. That is actually really uncomfortable […] and I feel like I have to make myself really small, so I don’t take up the whole seat […] So yes, sometimes I do [avoid travelling].” – Lea |
|  | Avoidance: Healthcare seeking  (-0.154) | 1 | Not seeking healthcare due to expected discrimination | “I can definitely put myself in her place [reaction to quote 1 about healthcare treatment]. I haven’t tried it, but I have also avoided seeing a doctor.” – Jane |
|  | Compensation:  Work  (0.800) | 4 | Feeling the need to compensate for EW and not confirming stereotypes by working harder | “I graduated 20 years ago and I actually think I have felt the need to do extra work all of my professional life because I’ve anticipated that people think I am lazy due to their prejudice [towards people with EW]. I simply cannot live with that prejudice because I really feel like I have done a lot of extra work compared to some of my colleagues. Maybe I would be lazier if I was normal weight (laughs).” – Kirsten |
|  | Compensation: Social life  (1.000) | 2 | Feeling the need to compensate for EW and not confirming stereotypes by being extra social and likeable | “I have felt the need to compensate [for my weight] in all sorts of different ways. I have been a smart ass, and people have thought I was really arrogant, but actually I was just really insecure right? I was so scared to be identified as a fat person. I was afraid that the others would notice my size and I would be an outcast like all the other fat people.” - Cecilia |
|  | Appearance  (1.000) | 1 | Feeling the need to compensate for EW and not confirming stereotypes by paying extra attention to ones’ appearance | “If you are fat and have bad teeth or greasy hair […] I think you are more prone to be stigmatised as a fat person. If you are clean and do not smell […] I don’t think it is as big a problem. […] Now that I am fat, I get my feet done or get my nails done and fix my eyebrows. These are some of the things I can still do to make my body look nice, because I know, body-wise, it’s not pretty.” – Mary |
| Mental well-being | Anxiety  (1.000) | 2 | Reported anxiety due to EW distress | “I have had quite a lot of anxiety about dying. For example, if I am in the cinema, I can get scared that I am dying […] but I am not actually afraid of death, I am afraid of causing a scene. Then I think to myself – okay, if I am dying, I just have to die quietly, so no one will notice. Then they can carry me out after the movie.” – Cecilia  “I can get a feeling of social anxiety sometimes when I go out, especially if it's people I've known once [and have not seen for a while], but I can also get it when I am meeting new people and the first thing they do is to look me up and down.” – Hellen |
|  | Social anxiousness  (1.000) | 7 | Feeling anxious or uncomfortable about social situations due to weight-related distress | “If I am invited to a big party where I don’t really know people then I think ‘Oh no, I don’t want to go’ because I feel like everyone is going to think ‘Oh my!’ [when they see me]. So, no, no, I don’t do that either.” – Hanna |
|  | Sadness  (1.000) | 2 | Reported sadness due to EW | “I have experienced being in a black hole, kind of, where I just lay there thinking ‘What am I supposed to do? Should I just end it, or should I try to move on? Is there anyone out there who can throw me rope, so I can get back up?’” – Simon |
|  | Loneliness  (1.000) | 1 | Reported loneliness due to EW | “I tried to explain how I felt to an ex-boyfriend and he didn’t understand. That’s when I realized that I have never actually had a person in my life that has understood how it felt to be me – ever! And then I started crying. I think I cried for the last 30 years because I have just been feeling so lonely.” – Cecilia |
|  | Disordered eating  (1.000) | 4 | Reported disordered eating due to EW distress | “One gets really sad and one’s self-esteem is just [thumbs down] and then one eats cake, chips and drinks soft drinks.” – Simon  “I was just interviewed about BED [binge eating disorder] the other day. […] I have also been treated for BED.” – Hellen |
|  | Aggression  (1.000) | 1 | Reported aggression due to EW distress | “I was arrested once because I resorted to violence [as a result of being harassed]. I talked to the police, and after hearing both sides of the story, they dropped the charges. I can understand why he reported me to the police, because I did resort to violence against him, but then again, he started it. So yes, it has been tough to have to look the other way, no matter what. It is easier to resort to violence and knock him out.” - Simon |
|  | Low self-esteem  (1.000) | 7 | Reported low self-esteem due to EW | “I had a boyfriend who said I was fat and stupid and then I thought ‘Yeah, maybe I am fat and stupid’. I mean, I don’t see myself that way, but then I just thought ‘Okay, I give up’. Then I became fatter and got a fat belly and fat thighs and droopy arms.” – Lea  “I hate going out. I mean, I am so big and what should I wear? I look like a cow.” – Hanna |
